# Physical model of serum supplemented medium flow in organ-on-a-chip systems

**DOI:** 10.1371/journal.pone.0322069

**Published:** 2025-06-17

**Authors:** Viesturs Šints, Jānis Cīmurs, Mihails Birjukovs, Ivars Driķis, Karīna Goluba, Kaspars Jēkabsons, Vadims Parfejevs, Una Riekstiņa, Gatis Mozoļevskis, Roberts Rimša, Guntars Kitenbergs

**Affiliations:** 1 Laboratory of Magnetic Soft Materials, University of Latvia, Riga, Latvia; 2 Faculty of Medicine and Life sciences, University of Latvia, Riga, Latvia; 3 Micro and Nanodevices Laboratory, Institute of Solid State Physics, University of Latvia, Riga, Latvia; Indian Institute of Technology Roorkee, INDIA

## Abstract

Creating a physiologically relevant shear stress in organ-on-a-chip (OOC) devices requires careful tailoring of microfluidic flow parameters. Currently, it is fairly common to use a simple approximation assuming constant viscosity, even for serum-based media. Here, we show that a popular nutrient solution (Dulbecco’s Modified Eagle Medium supplemented with Fetal Bovine Serum) requires a more complex treatment (i.e., is a non-Newtonian fluid), with observed shear stress values significantly greater than reported in literature. We measure the rheology of the solutions and combine it with a 3-dimensional flow field measurement to derive shear stress at the channel surface. We verify the experiments with numerical simulations, finding good agreement and deriving flow properties. Finally, we provide relevant expressions for the shear stress approximation, suitable for development of OOC devices with various geometries.

## Introduction

Organ-on-a-chip (OOC) systems are designed to mimic organ functionality in microfluidic devices [1]. Operation of such devices often involves fluid flow and interaction of this flow with organ cells within the device. Therefore, it is important to understand the properties of the fluid. A crucial consideration is whether the flow medium in the OOC device is Newtonian (in which case viscosity at a given temperature and pressure is given by a single parameter and does not depend on the fluid shear rate) or generalized Newtonian (fluid viscosity depends on the shear rate), which are a subset of non-Newtonian fluids. Shear rate is a measure of the rate of change of velocity over, in this case, the cross-section of a flow channel, with the shear rate distribution in generalized Newtonian fluids deviating significantly from that of Newtonian fluids.

Shear stress within a flow channel of a microfluidic device depends on both shear rate and viscosity, and is therefore likely to significantly vary if fluid viscosity depends on the shear rate. At the same time, shear stress is also associated with essential physiological effects [2], making it fundamental to multiple current and potential OOC applications. For example, in the case of blood vessels, shear stress can affect vascular morphogenesis, maturation and vessel permeability [3, 4]. Endothelial cells reorganize their cytoskeleton and adjust the composition of intercellular junction proteins according to the shear stress experienced [5, 6]. Similarly, in epithelial cells, certain intensity of fluid shear stress can cause a variety of phenotypical, metabolic and functional alterations, including changes in the permeability of the epithelial layer and cell maturation [7, 8]. In addition, forces due to shear stress are also involved in the pathophysiology of some disorders, such as obstructive pancreatitis: prolonged stimulation of a mechanically responsive ion channel PIEZO-1 by high shear stress is sufficient to induce changes in pancreatic stellate cells and cause organ fibrosis [9], while similar activation of acinar cells triggers pancreatitis [10]. With these crucial considerations in mind, the level of shear stress should be adjusted based on the type of cells it will affect. For cells that naturally experience shear stress, it is important to apply an optimal range to support their function. However, for cells that are sensitive to shear stress, it should be minimized to avoid detrimental effects. [11].

With this in mind, the determination of flow shear stress in OOC devices is clearly an important task. Relevant methods include the use of online tools offered by microfluidics companies [12], numerical simulations of fluid flow, and the use of a relation between shear stress and flow rate in a channel of known geometry (e.g. [13–15]). Using either of the methods requires knowledge of the medium viscosity, which means one must classify the fluid as Newtonian or generalized Newtonian. Note that relating the flow rate and shear stress via the Navier-Stokes equations, in the form used in the OOC research cited above, is only appropriate if the fluid is Newtonian.

To verify the fluid flow model assumed in the literature, we perform our experiments with a popular[16] commercial culture medium: Dulbecco’s Modified Eagle Medium (DMEM), supplemented with Fetal Bovine Serum (FBS). The viscosity of DMEM supplemented with various concentrations of FBS, depending on the shear rate, has previously been measured [17]. However, the author of the paper does warn about possible shear thinning behavior for conditions not considered in the study (which limits itself to a range of shear rate values that may entirely not cover those encountered in OOC applications) and recommends repeated measurements at lower flow rates.

Much of the reported research assumes that cell culture media, i.e., DMEM and FBS mixtures, are Newtonian. This is either explicitly stated (see Ref. [18] for example and Ref. [19] for discussion on rheology of bodily fluids and cell culture media), or implied by the use of a simpler, Newtonian fluid approximation of the Navier-Stokes equation (Ref. [15] for example) or the use of a single value for viscosity (Ref. [7] for example) – both of which are appropriate only for Newtonian fluids, or can be approximated as such within a relevant shear rate range. The last point is illustrated by looking at Ref. [13], where the authors refer to the viscosity provided in Ref. [17]. However, judging by the geometry and the specified flow rate values, the shear rate values appear to fall within a range where Ref. [17] indicates possible shear thinning. Experimentally, higher microcarrier concentrations have been observed leading to higher shear stress and the emergence of shear thinning behavior in cell culture media[20].

As the experimental measurement results presented in this paper will demonstrate, this nutrient solution is indeed non-Newtonian, and exhibits a shear thinning behavior. We show that a power-law model can be used to describe the viscosity of the medium. The flow velocity and shear rate values presented here should be relevant for a variety of applications, including OOC of intestinal and kidney cells [21].

Further, we will present measurements of flow fields within a channel of an OOC device, focusing on the shear thinning effects on the flow velocity distribution across the channel cross-section. We will introduce a mathematical description for the flow and present numerical modeling results, which are compared to the experimental data. Finally, we will use experimentally determined flow velocity and fluid viscosity to derive shear stress values at channel walls, allowing us to demonstrate the magnitude of the effect using the measured viscosity values, instead of relying on the data reported in the literature under the assumption that DMEM supplemented by FBS is a Newtonian fluid.

Note that the Results section contains only a brief description of experimental, mathematical and numerical methods, for context. Details are provided in the Methods and Materials section.

## Results

### Medium & its viscosity

**Fig 1:**
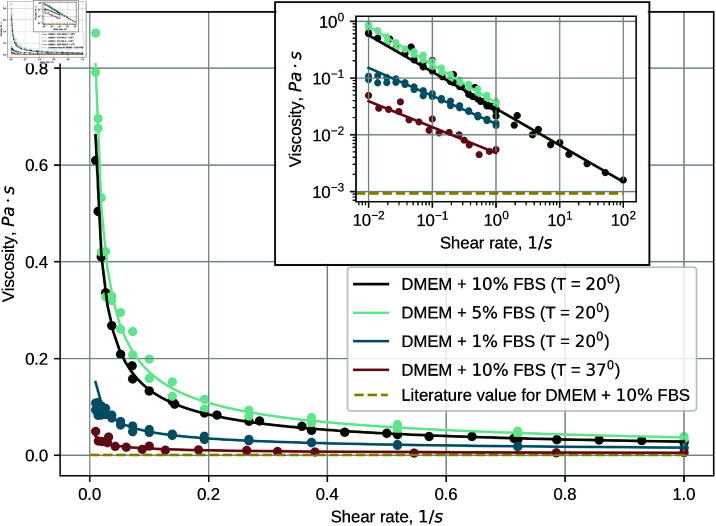
Viscosity of the medium over the shear rate range relevant to cell growth. The solid lines represent fits of experimental data to power law (5). Inset: Viscosity plots in log-log coordinates, showing the correspondence to the power law. Note that two additional measurements have been added to the DMEM + 10 % FBS data set, expanding the range of the data.

The substance used for experimental investigation was DMEM supplemented with a variable amount (1%, 5% or 10%) FBS and 1% penicillin/streptomycin (all supplied by *Gibco*). The viscosity of the medium was measured with a cone-plate rheometer, at room temperature (T=20^∘^C) and at T=137^∘^C. The dependence of viscosity on the shear rate is shown in Fig 1. Most of the results are from the measurements at room temperature, with the inset focusing on a measurement of DMEM + 10 % FBS at T=37^∘^C. The latter data set is the most relevant to actual cell cultivation. The dashed line in Fig 1 corresponds to the dynamic viscosity value of DMEM + 10 % FBS provided in [17], $\mu =9.3\times 10^{-4}\;Pa\;s$. It should be noted that similar values are also used in other papers, e.g. [7], and therefore this baseline will be referred to as the *literature value*.

An immediate observation is that, while the measured values do converge to the literature value as the shear rate increases, the viscosity at lower shear rates is much greater than the literature value. Note that the shear rate range shown in Fig. 1 contains values that we would encounter in the OOC device used in our experiments; please,see Supporting information (S1_Fig_shear_rate) for the shear rate data. The expanded data set in the inset demonstrates that the values we have measured approach the literature value at sufficiently high shear rates.

Clearly, none of the samples are Newtonian fluids. Instead, the viscosity decreases with the shear rate, which is known as shear thinning. For non-Newtonian fluids, the relation between shear rate and viscosity is given by the Ostwald-de Waele power law (5). The fit parameters for (5) are summarized in Table 2.

One can also note two trends: viscosity values increase with the FBS concentration, and the increasing temperature decreases the viscosity, as expected. As seen in the inset of Fig. 1. However, even at body temperature, the viscosity exceeds the literature value over the entire shear rate range.

### Fluid flow in the OOC device

**Fig 2:**
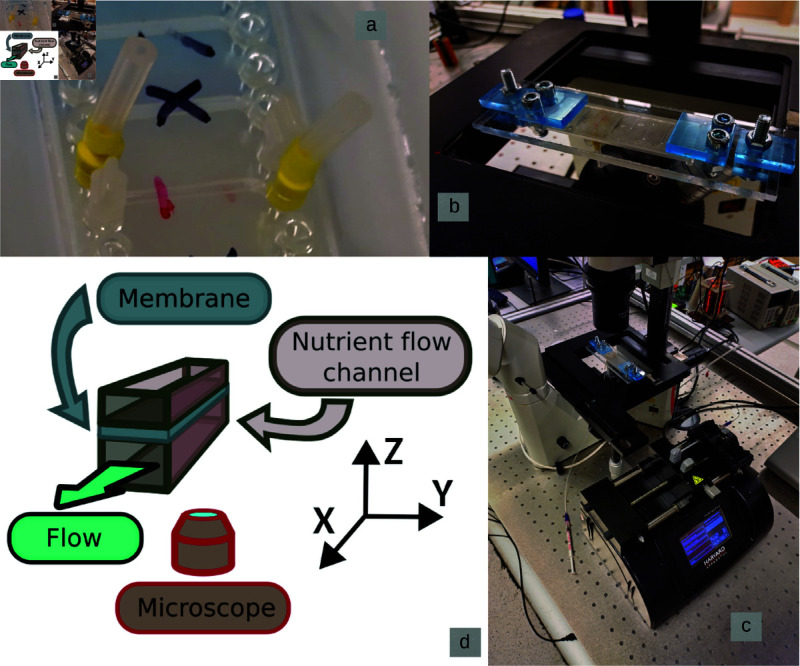
The experimental setup: (a) an array of channel pairs, the pair marked red has the inlet and outlet for one channel connected (the thicker, nutrient flow channel), and blocked for the other channel; (b) OOC device placed in a microscope; (c) the microscope with an OOC device and the syringe pump; (d) a schematic of the channel layout.

We used OOC chips with pairs of vertically stacked channels separated by a PET membrane. We examine the flow in one of the channels, 1.25 mm high and 1.2 mm wide, while the other, thinner channel, is filled with the same fluid, but its inlet and outlet are blocked. The flow is generated by a syringe pump, with a flow rate set to 4 or 8 μL/min. The flow rates correspond to the cell cultivation process reported with chips of similar geometry [22]. The chips are held in a microscope using a fixture. A schematic of the resulting geometry is shown in Fig. 2, where coordinate conventions are also introduced.

**Fig 3:**
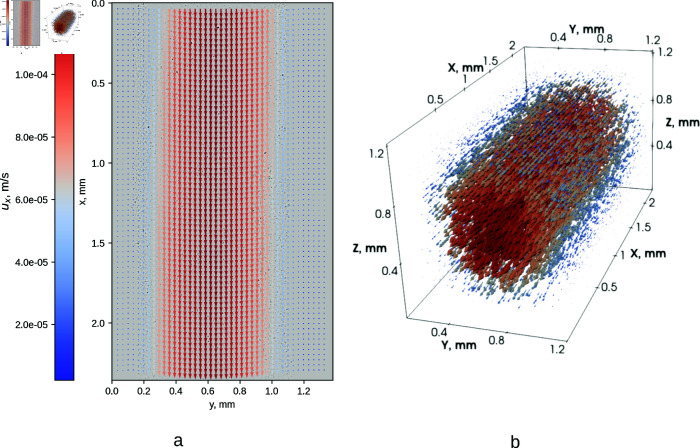
An example of a measured velocity field in the microchannel water at flow rate 4 μL/min). Left: velocity distribution at a given *z* coordinate value (z=0.5 mm). Right: a vector field $\vec{u}(x,y,z)$ assembled from a stack of plane measurements for the channel volume within the microscope field of view.

The flow field within the channels was measured using particle image velocimetry (PIV) at the microscope focus distance, giving us velocity distribution in (*y*,*x*) coordinates, at a single *z* coordinate, as shown in Fig 3a. We do this for every relevant *z*. Stacking these vector fields then gives us a flow field for the entire channel, as shown in Fig 3b.

**Fig 4:**
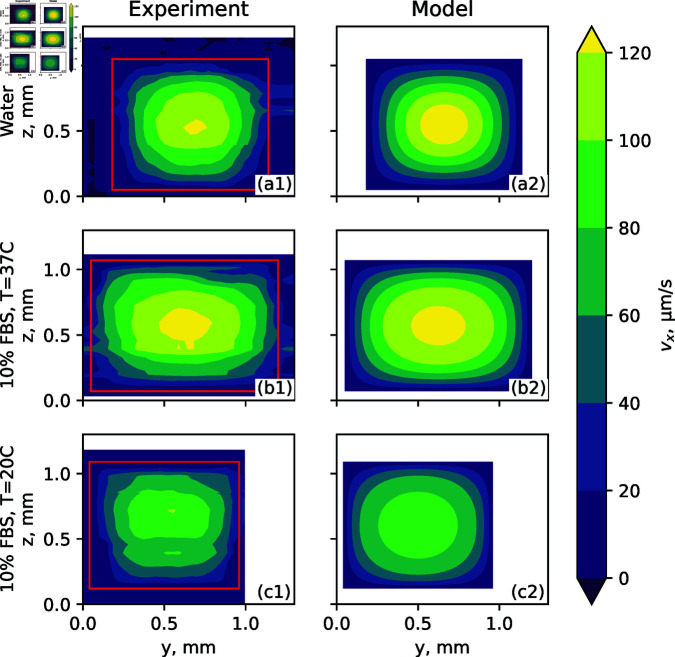
(a1), (b1), and (c1) – Flow velocity obtained using PIV; (a2), (b2) and (c2) – numerically calculated flow velocity using COMSOL. (a1) and (a2) – water at room temperature T=20^∘^C (*n* = 1); (b1) and (b2) – DMEM + 10% FBS at 37^∘^C (*n* = 0.5); (c1) and (c2) – DMEM + 10% FBS at 20∘C(*n* = 0.32). Channel boundaries are indicated with red lines (zero velocity boundary condition). Here *n* is the flow index in the power law (5).Different channels were used for different mediums.

Flow field measurements were performed for DMEM supplemented with all FBS concentrations considered in Fig 1, as well as water, for reference. Cross-sections of the average flow fields in (*y*,*z*) coordinates are shown in Fig 4 (a1), (b1), and (c1). The corresponding numerical modeling results are shown in Fig 4 (a2), (b2), and (c2), where the experimentally obtained dependence of fluid viscosity on shear rate has been employed. The samples represented here are water (Newtonian), DMEM supplemented by 10% FBS as a more extreme example of a shear thinning, and DMEM supplemented by 10% FBS at 37∘C, all at a 4 μL/min flow rate. The latter sample is the most representative of a medium that would be used in OOC. Different channels were used for different mediums. The uncertainty of the channel width and height is shown in Fig 4.

**Fig 5:**
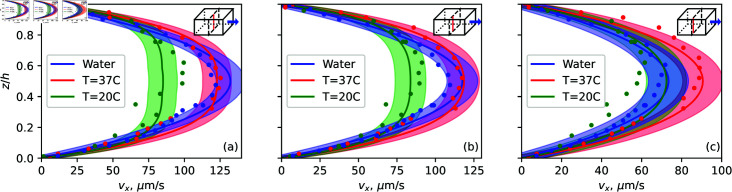
Velocity profiles obtained via PIV (dots) and COMSOL (solid lines) for 3 different fluids: water at room temperature T =20∘C (blue, *n* = 1), DMEM + 10% FBS at T =37∘C (red, *n* = 0.5), DMEM + 10% FBS at room temperature T =20∘C (green, *n* = 0.32), all at flow rate 4 μL/min. The profiles are compared in the middle of the channel (a), 1/3 of the channel width (b), and 1/6 of the channel width (c). Shaded regions are 10% error margins for COMSOL. *n* is the flow index in the power law equation (5).

As seen in Figs 4 and 5, the shear thinning behavior of FBS-supplemented DMEM results in a flatter flow profile at the center of the channel, with a less pronounced velocity peak at the center of the channel - with a tendency to a plug flow, where the velocity changes mostly near the walls of a channel and is nearly constant closer to the center. The shear rate distribution in the channel is therefore different from that in the case of a Newtonian fluid. This trend is observed in both the experimental and numerical results, and the velocity maxima are in reasonable agreement. The plug flow can be explained through an analogy to an extreme shear thinning example – Bingham plastic, which flows only when certain shear stress is reached. Due to symmetry, the shear rate and shear stress at the channel center would then be zero, and increase toward the boundaries. Flow can be observed only when a certain shear stress is reached, which can be close to the channel walls. And the middle part flows as a plug. Sources of measurement errors and discrepancies are discussed in the Methods and materials section. The red lines in Fig 4 represent the boundaries of the channel, identified as described in the Methods and materials section. The correspondence between theoretical and experimental values is further analyzed in Fig 5, where flow velocity profiles are compared at several *y* values. The experimentally obtained velocity profiles agree with the theoretical model qualitatively and, within the error margins, also quantitatively.

### Shear stress due to fluid flow

We have by now demonstrated that DMEM supplemented with various amounts of FBS exhibits shear thinning behavior, with a viscosity that is a function of the fluid shear rate, and is significantly larger at low shear rate values than previously assumed in the literature. We have also obtained flow velocity fields from within the flow channels in an OOC device, and demonstrated the effect of the shear thinning on the velocity distribution. Flow shear stress, being a product of shear rate and fluid viscosity, would be expected to be affected dramatically, compared to a Newtonian fluid with viscosity similar to that reported for DMEM supplemented by FBS at higher shear rates (or assuming independence of shear rate).

**Fig 6:**
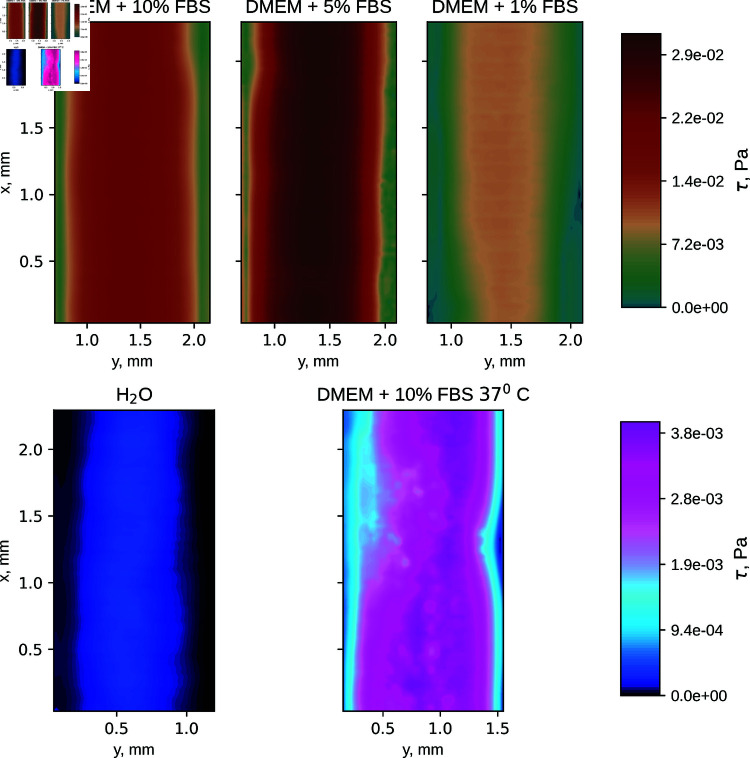
Shear stress at the membrane, which is the channel surface relevant for cell growth. Note the different color schemes in top and bottom rows, necessitated by the significant spread of of shear stress values. Flow rate 4 μL/min used in all measurements.

The shear stress field is derived from the experimental results using the velocity field obtained and the shear rate to shear stress relations. We focus on the values near the membrane (the top wall of the channel) where cell growth would occur. The experimental shear stress fields near the membrane are shown in Fig 6. These fields exhibit a wide range of shear stress values – note the different scales used for the top and bottom rows of Fig 6. The results most relevant for OOC research are for DMEM + 10 % FBS at 37 ∘C temperature (corresponding to the OOC working conditions) and a Newtonian fluid, water.

For reference, we also compare the experimental values to the results we would obtain if we were to assume the nutrient solution to be a Newtonian fluid. For this, we introduce the concept of *Newtonian DMEM + 10% FBS*. As we have shown here, this is not a physically valid model of the fluid – it is, however, currently the most common treatment in the literature; hence, we refer to it as the *literature model*. To obtain shear stress for this case, we note that all Newtonian fluids exhibit identical velocity distributions under the same boundary conditions in the channel. If DMEM + 10% FBS was Newtonian, it would have the same flow velocity distribution as water, so we use the velocity field measured for water as the result for a general Newtonian fluid. The viscosity is assumed to be constant and equal to $\mu =9.3\times 10^{-4}\;Pa\;s$. One way to describe this approach is – this is a description of DMEM + 10% FBS that we would arrive at by following the data in the literature. The maximum shear stress on the membrane given by this model is compared to the results obtained experimentally and numerically in Table 1, showing an order of magnitude difference between the literature model and the actual DMEM + 10% FBS values at 37∘C.

**Table 1:** Maximum shear stress values at the flow channel membrane where cell growth would occur: results from experimental data and numerical modeling. Note that the experimental values could be expected to have as much as 25% measurement error

Sample	$\tau_{max}$, ${dyne/cm}^{2}$	$\tau_{max}$, Pa	
	Experimental	Experimental	COMSOL
DMEM + 10% FBS	0.21	0.021	0.026
DMEM + 5% FBS	0.31	0.031	0.033
DMEM + 1% FBS	0.098	0.0098	0.013
H_2_O	0.0052	0.00052	0.00052
DMEM + 10% FBS 37∘C	0.046	0.0046	0.0039
Literature	0.0054	0.00054	0.00054

From the results of the theoretical and numerical modeling, we see that the shear stress τ exerted by the fluid is a power law function of the flow rate *Q*:


$$\tau =K_{\tau }Q^{\frac{1}{n}}$$

where *n* is the flow index in (5) and $K_{\tau }$ is a parameter depending on the channel and fluid. In a Newtonian fluid (*n* = 1), shear stress is proportional to the flow rate. This and other scaling laws found in the numerical simulations section allow us to compare different experimental conditions.

As seen from the simulation results in Figs 4 and 5, the velocity profile due to shear thinning has a larger plateau in the middle of the channel. This results in a wider region of higher shear stress in the channel wall, as seen in Fig 6. From simulation results for a square cross-section, we know that the region of the wall with shear-stress not less than 90% of the maximal value is in the middle 32% of wall area for the Newtonian fluid; 47% for the DMEM + 10% FBS at 37∘C (body temperature); 52% for the DMEM + 10% FBS at 20∘C (room temperature). The area of the high shear region can be increased not only by switching from Newtonian to a shear thinning fluid, but also by using a much wider rectangular channel.

## Discussion

We have shown that DMEM supplemented by FBS exhibits shear thinning behavior with viscosity values diverging significantly at lower shear rates from a value referred to in multiple literature sources – this constant, shear rate-independent value is only appropriate at shear rates in excess of those relevant to the OOC system used in our research. Reviewing the typical shear stress values used in OOC research [11, 21] and comparing them with the shear rate to shear stress relation seen in our measurements leads us to believe that a significant portion of experimental research in the field deals with shear rates in the range of $1\times 10^{-2}\;s^{-1}$ to $1\;s^{-1}$, where shear thinning should be considered. This contradicts a significant amount of published research. At the same time, this conclusion is in agreement with Ref. [17], and agrees qualitatively with the conclusions in Ref. [23].

Investigation of the rheological properties of the nutrient solution has shown that the viscosity of the solution increases with the concentration of FBS. This suggests that FBS determines the shear thinning behavior of the solution. However, our experimental data presented here do not yet allow one to conclude this definitively. Quantification of the role of BS ingredients (proteins, lipids, vesicles, and others) in rheological parameter formation would require content and structure analysis of the substance, which is beyond the scope of this article. However, this is not unexpected, with analogy to systems where shear thinning could be expected – rod and fiber suspensions [24], and polymer solutions [25]. The viscoelastic properties of serum-based cell culture medium remain a topic for future research that could lead to a deeper understanding of the rheology of these solutions.

We have developed a method for measuring the flow velocity field directly in an OOC device, and have shown a correspondence between the experimental and theoretical velocity values. The measured velocity profiles are within expectations for a shear thinning behaviour, and provided us with the data required for shear stress calculation (viscosity parameters and the velocity field).

The shear thinning effects observed in the flow of nutrient solution and the corresponding increase in shear stress are among the most significant results of this article. Shear stress measured in DMEM supplemented by 10 % FBS at 37∘C exceeds both the values seen for water and calculated using the viscosity value commonly found in literature by an order of magnitude. The results suggest that the FBS content in the solution could strongly influence its rheological properties, particularly the shear stress exerted on the channel walls. The considerable discrepancy itself is noteworthy, even if the actual values could not be repeated at different geometries and flow rates. All of the above constitutes a strong argument against the use of (in this context) oversimplified shear rate-independent viscosity values and Newtonian fluid models for serum solutions.

We suggest the following approximate formula to calculate the flow rate *Q* necessary to obtain the shear stress τ at the base of a channel with height *h* smaller than width *w*

$$Q=\frac{h^{2}w\cdot n}{2(1+2n)}{\left(\frac{\tau }{K}\right)}^{\frac{1}{n}}\cdot \frac{1-\frac{192}{\pi^{5}}\cdot \frac{\cosh(\pi w/h)-1}{\sinh(\pi w/h)}}{1-\frac{8}{\pi^{2}\cosh((\pi w)/(2h))}}$$ (1)

where *K* (in units ${Pa/s}^{n}$) and *n* are power-law fluid parameters. *K* and *n* values used in this study are available in Table 2. The given formula is a combination of flow in a rectangular channel with small height (9) and a formula for Newtonian flow in a rectangular channel with variable dimensions[26]. If the formula (1) is used, the error for the shear stress will be less than 3% (See comparison to simulated data in supporting information S4 Fig error).

**Table 2:** Fit results for *K* and *n* values in (5) for the experimental rheometry data shown in Fig 1.

Sample	K	n
DMEM+10% FBS	0.028	0.31
DMEM+5% FBS	0.037	0.33
DMEM+1% FBS	0.016	0.51
DMEM+10% FBS (37∘C)	0.005	0.54

## Methods & materials

### Theoretical model

**Fig 7:**
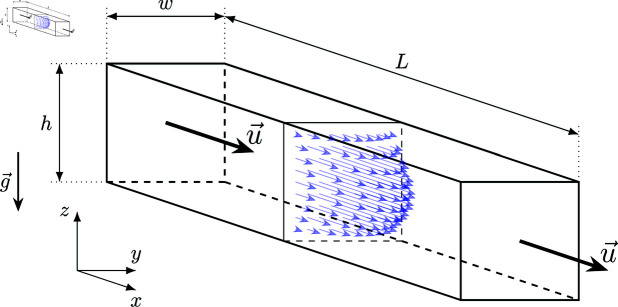
A schematic of the fluid flow in a rectangular channel with width w (y-axis), height h (z-axis) and length L (x-axis).

The fluid velocity field can be calculated using the Navier-Stokes equation. In microfluidics, the channel dimensions are ~ℓ=1 mm, the flow rate is <Q=10 μL/min, and the kinematic viscosity is $\nu =\mu /\rho \gg 1\times 10^{-6}\;m^{2}\;s^{-1}$ (water), with the Reynolds number Re≪Q/νℓ=0.17. This is known as the creeping flow or the Stokes flow, with negligible inertial effects. Therefore, here the fluid flow is described by the Stokes equation [27], [28, Chapter 4.8]

$$\nabla p=\nabla \cdot \left(\mu \nabla \vec{u}\right)$$ (2)

and the continuity equation for an incompressible fluid

$$\nabla \cdot \vec{u}=0\text{ ,}$$ (3)

where $\vec{u}$ is the velocity field, *p* is the pressure field, and μ is viscosity; $\nabla \vec{u}$ is the strain rate tensor, and $\mu \nabla \vec{u}$ is the shear stress tensor. The protein polymer chains can be extended by the flow in the *x*-direction (Fig 7), but that should not influence shear in the *yz* plane. If the polymer chirality is not pronounced, clockwise and counterclockwise rotation should be equivalent. Therefore, we assume an isotropic fluid, with an isotropic relation between the shear rate and shear stress tensors, meaning μ is a scalar.

Due to the symmetries of the problem, steady fluid flow in a channel with a constant cross-section can be expressed in Cartesian coordinates (please see axis definitions in Fig 7) using (2):

$$\frac{\partial }{\partial y}\left(\mu \frac{\partial u_{x}}{\partial y}\right)+\frac{\partial }{\partial z}\left(\mu \frac{\partial u_{x}}{\partial z}\right)=\frac{\delta p}{L}$$ (4)

With the pressure gradient only along *x*-axis for a constant cross-section the flow is unidirectional,


$$u_{z}=u_{y}=0$$

and the continuity equation (3) enforces the fully developed flow:


$$\frac{\partial u_{x}}{\partial x}=0$$

The pressure gradient is constant


$$\frac{\partial p}{\partial x}=\frac{\delta p}{L}<0$$

and negative (since *u*_*x*_>0), where δp is the pressure difference between the ends of the channel with length *L*.

The shear thinning behavior of a biological fluid containing many long protein chains can be described using the Ostwald–de Waele power law [29]:

$$\mu =K{\dot{\gamma }}^{n-1}$$ (5)

where *K* and *n* are the flow consistency and the flow index, respectively, determined experimentally, and

$$\dot{\gamma }=\sqrt{{\left(\frac{\partial u_{x}}{\partial y}\right)}^{2}+{\left(\frac{\partial u_{x}}{\partial z}\right)}^{2}}$$ (6)

is the second invariant of the strain rate tensor. Note that *n* = 1 corresponds to a Newtonian fluid; if 0<*n*<1, one has a shear-thinning behavior, common for solutions with polymer chains; *n*>1 yields a shear-thickening behavior, a behavior observed in granular matter. Here, we focus on a shear-thinning fluid with 0<*n*<1.

Power-law fluids come from a family of non-Newtonian fluids called generalized Newtonian fluids, where the viscosity depends on the shear rate. A power-law fluid has only two phenomenological parameters, *K* and *n*, and is limited to a specific intermediate range of shear rates. There exist more sophisticated models of Generalized Newtonian fluid, e.g., the Carreau viscosity model [30, 31], which works better for a largeshear rates and does not have an infinite viscosity at zero shear rate. However, this and other more advanced models contain more parameters, which could result in over-fitting the noisy experimental data instead of capturing the essential physics. Therefore, we use the power-law relationship (5), which is reasonably in good agreement with the rheometer data.

#### Analytical solution in a rectangular channel with small height.

In our experiments, the channel is rectangular. Generally, a velocity field of a power-law fluid in a rectangular channel can not be obtained analytically. Therefore, numerical simulations are necessary. However, for a channel with a small height, an analytical solution exists. If the channel width is much larger than the height, the following velocity distribution between parallel plates can be obtained[30]:

$$u_{x}(z)=\frac{n}{n+1}{\left(\frac{\left|\delta p\right|}{KL}\right)}^{\frac{1}{n}}\left({\left(\frac{h}{2}\right)}^{\frac{1}{n}+1}-{\left|\frac{h}{2}-z\right|}^{\frac{1}{n}+1}\right)$$ (7)

The corresponding flow rate is

$$Q=w\int_{0}^{h}u_{x}(z)\;\text{d}z=\frac{h^{2}w\cdot n}{2(1+2n)}{\left(\frac{\left|\delta p\right|h}{2KL}\right)}^{\frac{1}{n}}$$ (8)

And the corresponding shear-stress on the upper and lower walls of the channel produced by the power law fluid is:

$$\tau =\frac{|\delta p|h}{2L}=K{\left(\frac{2Q}{h^{2}w}\left(2+\frac{1}{n}\right)\right)}^{n}$$ (9)

Here, *n* = 1 yields a Newtonian fluid with a Hagen–Poiseuille parabolic velocity distribution, with K→μ [32–34]. One can see from (9) that shear-stress τ is linear in flow rate *Q* only if the fluid is Newtonian (*n* = 1). In a power-law fluid, shear-stress τ scales non-linearly with *Q*.

### Numerical simulations

Fluid flow was simulated using a finite element method using the *COMSOL Multiphysics*^®^ (COMSOL) software [35]. The problem was solved for a 2D (two-dimensional) rectangular geometry using the coefficient form PDE. The solved equation is (4) with an isotropic coefficient given by (5). Dirichlet boundary conditions are set for all boundaries. The channel dimensions correspond to the experiments. The velocity field was initialized as follows


$$u_{x}(y,z)=\frac{4u_{max}\cdot yz(w-y)(h-z)}{w^{2}h^{2}}$$

where *u*_*max*_ is the maximal flow speed of the fluid flow from the experiment with similar flow rate. Extra fine physics-controlled mesh was used(a triangular mesh with the average side length 0.02 mm). The problem was solved using a stationary solver. The *K* and *n* values chosen according to Table 2 and the pressure gradient $\frac{\delta p}{L}$ is chosen to match the flow rate with the experiments using the scaling laws presented below.

The results in a simulated channel and a channel with a small height yield the following scaling laws:

- Shear stress τ is proportional to the pressure gradient δp/L and the linear dimension of the channel *h*: $\tau =C_{s}$ · *h* · δp/L, where *C*_*s*_ is a dimensionless constant dependent on the shape of the channel.
- The velocity profile *u*_*x*_(*y*,*z*) is proportional to the flow rate *Q* and inversely proportional to the cross-section area *A*: *u*_*x*_(*y*,*z*) = *Q*/*A* · *f*_*s*_(*y*,*z*), where *f*_*s*_(*y*,*z*) is a dimensionless function dependent on shape.
- Shear stress τ is a non-linear function of the flow rate *Q* and the linear dimension of the channel *R*: $\tau =K\cdot C_{\tau }\cdot (Q/R^{3}{)}^{\frac{1}{n}}$, where $C_{\tau }$ is a shape-dependent dimensional parameter and the values *K* and *n* are the viscosity parameters (5).

These scaling laws were tested using COMSOL for rectangular channels with different cross-section aspect ratios.

The obtained flow profile comparison with experimental results of two different flow rates can be seen in Supporting information S3 Fig. That demonstrates, that velocity is proportional to the flow rate.

### Fabrication & geometry of the OOC device

The chips have a vertically stacked design with the top channel height of 1.25 mm, designated for epithelial cells, and the bottom channel height of 0.20 mm, for endothelial cells. This distinction refers to the intended uses of both channels, whereas in the experiments described here, no cells are present in either of the channels. Both channels have an overlap area of $18\;{mm}^{2}$, with channel widths of 1.2 mm and 1.0 mm, respectively. A schematic of the resulting geometry is shown in Fig 2.

A syringe pump is used to generate flow in the larger of the channels, while the smaller channel is filled with the same fluid that is used for flow, with blocked inlets and outlets. The channels are separated by a 3 μm porous polyethylene terephthalate (PET) membrane (3 μm pore size, $0.8\times 10^{6}\;{pores/cm}^{2}$ and 5.7% porosity, manufactured by *it4IP*, Belgium).

Microfluidic devices are OSTE-based chips. OSTE, introduced in 2011[36], is an alternative to PDMS that has been used since the 1990s in microphysiological systems [37], OOCs, and other microfluidic devices, e.g, to model flow effects on plant roots [38]. The chips were designed in *Solidworks* (*DS Solidworks Corp.*, USA) and fabricated using the off-stoichiometry thiol-ene (OSTE) and cyclo-olefin copolymer (COC). We used COC mini luer port microscope slides for the top channels and standard COC microscope slides for the bottom layers (both by *Microfluidic ChipShop*, Germany). The master molds for the OOC channels were 3D printed using a masked stereolithography (MSLA) printer (*Zortrax Inkspire*, *Zortrax*, Poland) and ivory resin. The master mold was cleaned in an ultrasonic bath with isopropyl alcohol for 10 minutes, blow-dried with nitrogen, and then fully cured with UV light ($6.6\;{mW\;cm}^{-2}$ for 30 minutes), followed by thermal treatment at 60∘C for 48 hours. PDMS was then mixed in at a 10:1 ratio, poured into the molds, and cured at 60∘C overnight.

For device fabrication, an OSTE 322 mixture (*Mercene Labs*, Sweden) was prepared and processed in a *Thinky* mixer at 750 rpm for 5 minutes in theboth mixing and defoaming modes. The mixture was then degassed in a vacuum desiccator for approximately 20 minutes. The surfaces of the COC slides were oxidized for 2 minutes using a plasma asher (*GIGAbatch 360 M, PVA Tepla*, USA) at 600 W with an oxygen flow rate of 800 sccm. The assembly of the device is finalized by injecting the OSTE mixture into the PDMS molds for each side, and exposing them to UV light using a *Suss MA6* mask aligner (850 mJ bottom layer, 925 mJ top layer). The COC slide with the exposed OSTE was then pressed against the membrane, and cured on a hot plate at 60∘C for 1 hour, with PTFE films and a 2 kg weight pressing the devices onto the hot plate. After a similar preparation of the other layer, the two layers were aligned using an adapted alignment tool and pressed together to remove air pockets. The assembled OSTE/COC hybrid device was compressed from both sides and cured in an oven at 60∘C overnight.

### Viscosity measurements

The rheological properties of the fluids were measured with a *Anton Paar MCR 502* rheometer, in a cone plate mode, with a constant shear rate throughout the sample. Peltier heating was used for temperature control, with measurements performed at room temperature (T=20∘C) and T=37∘C.

The mixtures of DMEM and all concentrations of FBS used here were found to exhibit a significant startup viscosity, with up to 2000 s of probe rotation required for the viscosity to become steady for a measurement at a constant shear rate. An initialization procedure with 2000 s of rotation at $1\;s^{-1}$ shear rate was performed prior to viscosity measurements. We assume that this replicates the conditions in the substance flow within the tubing leading to the OOC device, and that it is therefore the stable plateau viscosity, rather than the transient startup value, that is relevant to the flow in the OOC device.

The measurements involved subjecting the sample fluid to rotation of the conical probe, with each measurement point corresponding to a 160 s measurement of the viscosity of the fluid at a given shear rate. Each sample was tested for a range of shear rates, focusing on measurements in the $\dot{\gamma }=0.01-1\;s^{-1}$ range. The overall range of shear rates covered experimentally was $\dot{\gamma }=0.01-100\;s^{-1}$. Measurements at shear rates below $\dot{\gamma }=0.01\;s^{-1}$ were unreliable.

To estimate repeatability of the results, measurements were made with different variations of the samples, including different samples with the same FBS concentration, repeated measurements for the same sample, including a refill of the rheometer, and measuring the same sample over consecutive days. We provide an example in Supporting information (S2 Fig). These measurements yield a 20% error estimate for parameter *K* and 9% for parameter *n* in (5). All viscosity values used in the experimental analysis are taken from the sample used in the respective experiment, with no more than 24 h passing between the viscosity measurement and the flow experiment.

Repeated simulations with different measured *K* and *n* values show that influence on the velocity profile is small for constant flow rate: the maximum velocity error is 2.5% and the maximum shear rate error is 7%; but the influence on shear stress is larger: shear stress error is 25%

### Particle image velocimetry & image acquisition

The PIV method is a type of image correlation velocimetry. The essence of the method involves taking two images of tracer particles within a fluid in quick succession, and measuring the displacement of these particles between the two images. This provides information on the velocity field of the fluid.

We use fluorescent 1.94 μm *Nile Red* particles (*Spherotech FH-2056-2*) mixed into the DMEM and FBS solution (or water), and subjected to ultrasound treatment. Viscosity measurements after tracer particle integration indicated that the solution properties were unaltered. We found that there was a risk of sedimentation for larger tracer particles, which limited the maximum experiment runtime.

Measurements were performed using an inverted microscope (*Leica DMI3000B*) with a 4× magnification objective, and a PIV setup by *Dantec Dynamics*, including the proprietary imaging software. A dual power 50-50 2×50 mJ, 532 nm laser was used to induce the tracer particle fluorescence prior to image acquisition. The pairs of images were acquired at 0.06 ms intervals with a 50 Hz frequency.

For image acquisition, the microscope is focused on a point with a user-defined *z* coordinate, following the definitions in Fig 2. The first point is typically placed below the lower wall of the channel to ensure that it is properly identified. Similarly, the last measurements are made at the *z* values above the channel and inside the membrane. A sequence of 100 pairs of images is acquired. This constitutes a measurement for one *z* value, or one flow layer. Subsequently, the microscope focus is changed to a different *z* value, and the next 100 image pair sequences are acquired, giving us the subsequent flow layer. The shift in microscope focus between two layers is δz=33 μm, although δz=66 μm was frequently used for the central areas of the channel. This typically resulted in 16-25 layers per full channel measurement. The images were taken near the middle of the channel *x* axis, as far as possible from the flow inlets and outlets.

The setup is enclosed in a heat insulating casing for temperature control. As in viscosity measurements, velocimetry is performed at room temperature and T=37∘C. The temperature inside the casing is controlled by a heater and a pair of *DS18B20* temperature sensors with an *Arduino* board. One of the sensors is placed near the chip, and the other between the chip and the syringe pump to ensure temperature uniformity within a δT=±2∘C error.

### Image processing

Processing of the obtained images is crucial for applying the described PIV method for velocity field measurements. Image quality is affected by the suboptimal (from the point of view of PIV analysis) choice of the tracer particles. However, the primary challenge involves discriminating the observed particles by the layer of flow in which they are a part. While adjustment of microscope focus brings one flow layer into focus, particles at different *z* values can still contribute to the signal, either as background luminescence or discernible entities moving at the velocity of their respective layer. Therefore, preprocessing of the raw images to enhance the signal-to-noise and contrast-to-noise ratios (SNR/CNR, respectively) is required to obtain reliable results.

Two approaches are used for image processing. The first involves a set of fairly standard procedures. Image gamma correction and histogram stretching are used to enhance the visibility of the tracer particles, followed by a successive application of low-pass and high-pass filters. All image transformations are performed in *Python*. The code is provided in *GitHub*: ViestursSints/ImageProcessing4PIV. In the other approach, image processing is performed using *Wolfram Mathematica* – the code is open-source and is available on *GitHub*: Mihails-Birjukovs/OOC_particle_flow_CNR_boost_for_PIV. This approach is explained in more detail in the following chapters.

The raw images are $1344\times 1024\;{px}^{2}$ 16-bit grayscale TIFs, with ~9 pixels per particle (PPP). Their field of view contains the fluid flow channel and the surrounding background, so channel area segmentation is performed. For this, the minimum pixel-wise temporal projection for the image sequence is computed, and the resulting image is inverted. To enhance channel wall CNR, soft color tone map masking (SCTMM) is applied [39–41], and then the initial channel wall mask is obtained via local adaptive binarization [42], with the local thresholding radius given by the median of image dimensions times a control parameter. To remove small-scale segmentation artifacts, a small-radius Gaussian filter is applied with subsequent re-binarization with the Otsu method [43], followed by morphological closing with a disk kernel [44]. Finally, to eliminate any remaining artifacts at the channel boundary, morphological dilation (disk kernel) [44] is applied to slightly extend the boundary, and the non-border segments are removed from the final mask.

The images are automatically cropped to the channel boundaries and the background correction of the image is performed as follows: each image in a sequence is inverted, divided by the mean image of the sequence (a flat-field correction, FFC, with mean reference), inverted again, and then the color tone mapping (CTM) is applied [45]. This correction method efficiently flattens the image luminance distribution and removes vignetting at the channel boundaries, as well as eliminates image artifacts due to reflections from the channel walls. Then, the resulting images are multiplied by an inverted channel mask to isolate the particle flow region for PIV.

Corrected images typically still exhibit a rather low CNR because of haze and dynamic large-scale features moving with the particle flow (essentially, correlated noise). Therefore, dehazing is performed first via two iterations of the following: an input image is inverted, normalized, then CTM, normalization, and referenceless FFC [46] are applied, followed by image inversion. Afterward, correlated noise and large-scale textures are removed using the non-local means masking (NMM) method [40, 41]. Both operations significantly boost the particle CNR, but the SNR is slightly reduced, so Perona-Malik anisotropic diffusion [47, 48] is applied to boost the image SNR while preserving particle CNR. Finally, to better resolve finer particles, CNR is boosted further by applying 15 iterations of the following: invert input images, apply CTM, invert the result. Optionally, particle binary masks can be obtained by applying Kapur’s segmentation algorithm [49], followed by boundary segment removal and size thresholding. The resulting filtered images can be used as PIV input. Compared to the other approach, this more advanced method was found to provide a better distinction for particles belonging to a particular flow layer, and sharper channel edge detection.

### Measuring the velocity field

PIV analysis is performed after image processing. The results shown here were obtained with the *OpenPIV* software for *Python*, although analysis using the *PIVlab* plugin for *MATLAB* was also performed for most of the relevant experimental data. Both methods yielded similar results. The PIV resolution (the smallest interrogation window size) was set to 32 px, resulting in a velocity field resolution δy=37 μm, roughly equal to the distance between flow layer measurements (δy≈δz).

The physical dimensions of the channel are determined either by the point where flow velocity drops to zero (the first and last layers to have nonzero velocities detected in them are assumed to be the layers near the top and bottom walls, while side walls can be identified by the velocity distribution by *y* reaching zero) or by using the channel masks obtained by image processing (side walls only).

In an attempt to estimate the precision of flow measurements, two key sources of error have been identified: fluid flow fluctuations and inconsistencies in the channel. The former is likely due to bubble formation, channel blockage, or some other defect along the flow path. As the flow fields corresponding to each *z* are obtained in succession, such fluctuations would affect one or several layers of measurement, effectively translating into spatial defects seen in the data. In practice, this is the primary source of the jagged edges of the velocity contours seen in Fig 4. From these deviations, we have observed that the velocity deviation reaches 7% of the maximum velocity value. Analysis of the 100-image sets for each measurement yields a standard deviation value of 4 × 10^−5^ m/s. However, this may not always be relevant, as the period of fluctuations may exceed the sampling period. Comparing different measurements at the same flow rates, where possible, gives us an error of around 15%.

Inconsistencies in channel geometry are another major source of discrepancy between theory and experiment. The theoretical/numerical model assumes the channel is straight, with a rectangular cross-section. In experiments, the channel walls are slightly jagged, and the channel cross-section is only approximately rectangular. Due to channel width variations and the fact that the top and bottom walls are not visible, the positions of the walls and the area of the cross-section of the channel will not be consistent for all *x* values when averaging over the measured flow field. We estimate the error of the channel width at ~5% of the average width.

Another noteworthy geometric inconsistency is the potential interference of the membrane with the flow. The unused channel filled with fluid should stop any mass flow through that channel, but this is not guaranteed. Our experimental method of measuring the mass flow does not allow us to observe the flow through the membrane if such a flow was present. An approach to estimate the influence of the membrane is to compare flow velocities measured near the solid bottom wall and near the membrane. At a distance of 0.033 mm from the last negligible-velocity flow layer at either end, the difference between measured velocities tends to be ~10%, with a maximum of ~30% for the DMEM+10% FBS sample. Given how close the deviations are to the measurement errors, and the possibility of other mechanisms affecting the flow symmetry (e.g., inlet placement), we cannot conclude whether the membrane influences the flow. This could be a subject for a separate study.

## Supporting information

S1 FigShear-rate in a square channel.Simulated data of the shear rate $\dot{\gamma }$ (6) in the middle of the channel (y=0.5 mm) for a channel with a square cross-section of size 1 mm × 1 mm. The flow rate in the channel is Q=4 μL/min. It can be seen that shear rate does not exceed $\dot{\gamma }=1\;1/s$ for all fluids discussed in this article: Newtonian fluid (Water, blue line) (*n* = 1); power-law fluid with *n* = 0.54 (DMEM+10% FBS at 37∘ C and DMEM+1% FBS at room temperature, red line); power-law fluid with *n* = 0.31 (DMEM+10% FBS and DMEM+5% FBS at room temperature, green line). *n* values correspond to power-law exponent (5). The bump near the center, pronounced by the red line, is due to nonphysical infinite viscosity at zero shear rate of the Ostwald formula (5). This bump has inessential influence on the flow profile and shear-rate on the wall. The less pronounced bump is by the green line.(TIF)

S2 Fig Viscosity measurement data. Viscosity measurements data for DMEM + 10 % FBS. Here we have reviewed three samples, performing measurements multiple times (denoted "runs"), including a refill of the same sample and measurements performed on different days. Ostwald-de Waele equation was fit to the entire ensemble of data points. A deviation 20% from the fit line is included as a measure of the repeatability of the results.
(TIF)

S3 FigComparison between flow rates.Here we show the experimental velocity values of the DMEM + 1% FBS sample in the middle line of the channel for two different flow rates Q=4 μL/min and Q=8 μL/min. We demonstrate here that increasing the flow rate two times, the fluid velocity increases two times. The model predicts the same shape of the velocity distribution for both flow rates.(TIF)

S4 FigError estimate of proposed formula.Here we show how large is the error of the proposed formula (1). The graph shows how the shear stress used to calculate flow rate $\tau_{\text{calculated}}$ using formula (1) differs from shear stress obtained using simulations $\tau_{\text{simulated}}$ for rectangular channel. The graph shows that shear stress in the channel will be higher than used in the formula by the amount which does not exceed 3%.(TIF)
