# Supplementary figures and images for: Physical model of serum supplemented medium flow in organ-on-a-chip systems
Source: PLoS One. 2025 Jun 17;20(6):e0322069. doi: 10.1371/journal.pone.0322069 (PMC12173184; doi:10.1371/journal.pone.0322069)

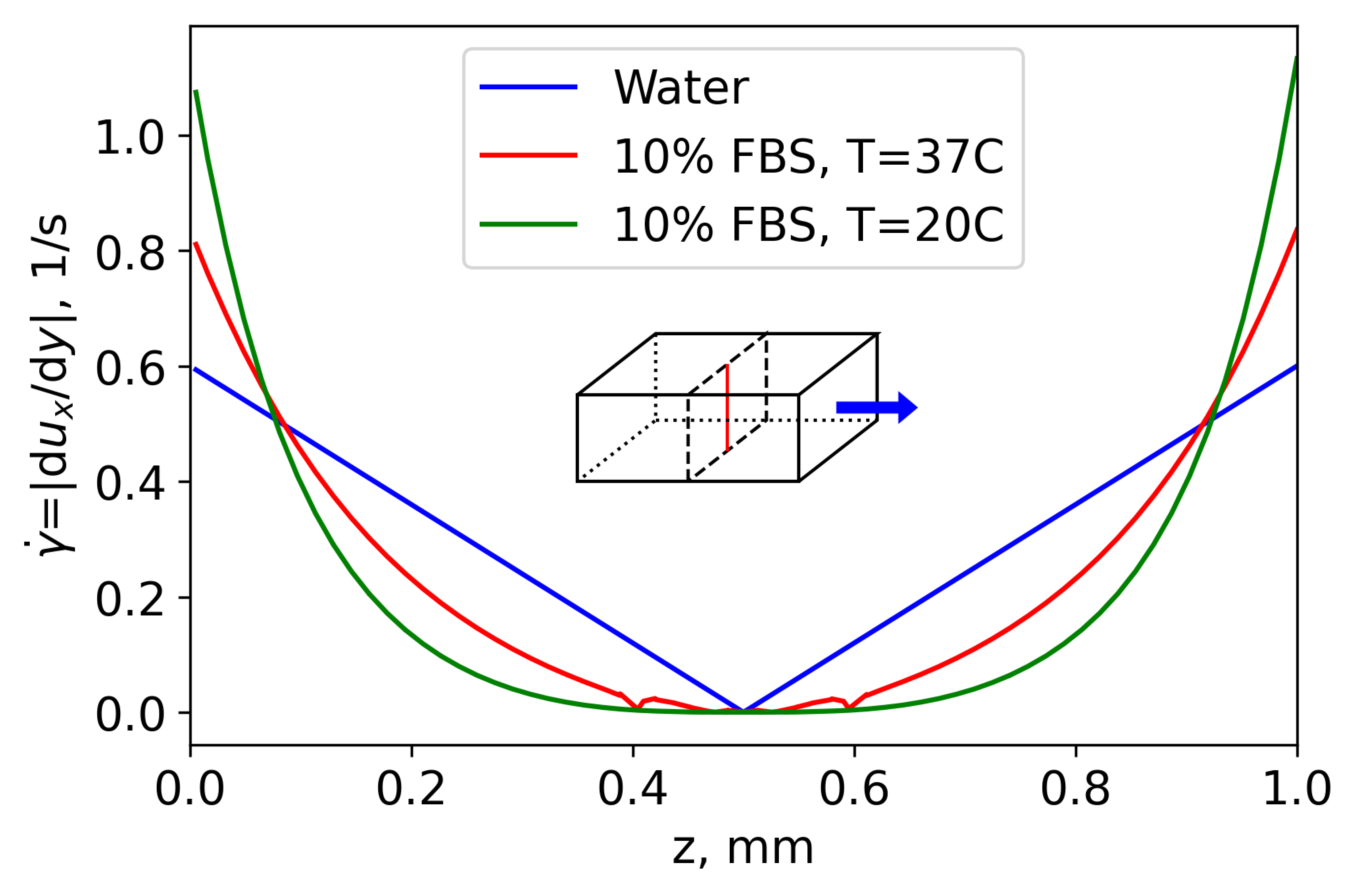

Supplement: S1 Fig — Simulated data of the shear rate γ˙ (6) in the middle of the channel (y=0.5 mm) for a channel with a square cross-section of size 1 mm × 1 mm. The flow rate in the channel is Q=4 μL/min. It can be seen that shear rate does not exceed γ˙=1 1/s for all fluids discussed in this article: Newtonian fluid (Water, blue line) (n = 1); power-law fluid with n = 0.54 (DMEM+10% FBS at 37∘ C and DMEM+1% FBS at room temperature, red line); power-law fluid with n = 0.31 (DMEM+10% FBS and DMEM+5% FBS at room temperature, green line). n values correspond to power-law exponent (5). The bump near the center, pronounced by the red line, is due to nonphysical infinite viscosity at zero shear rate of the Ostwald formula (5). This bump has inessential influence on the flow profile and shear-rate on the wall. The less pronounced bump is by the green line. (TIF) [file pone.0322069.s001.tif]

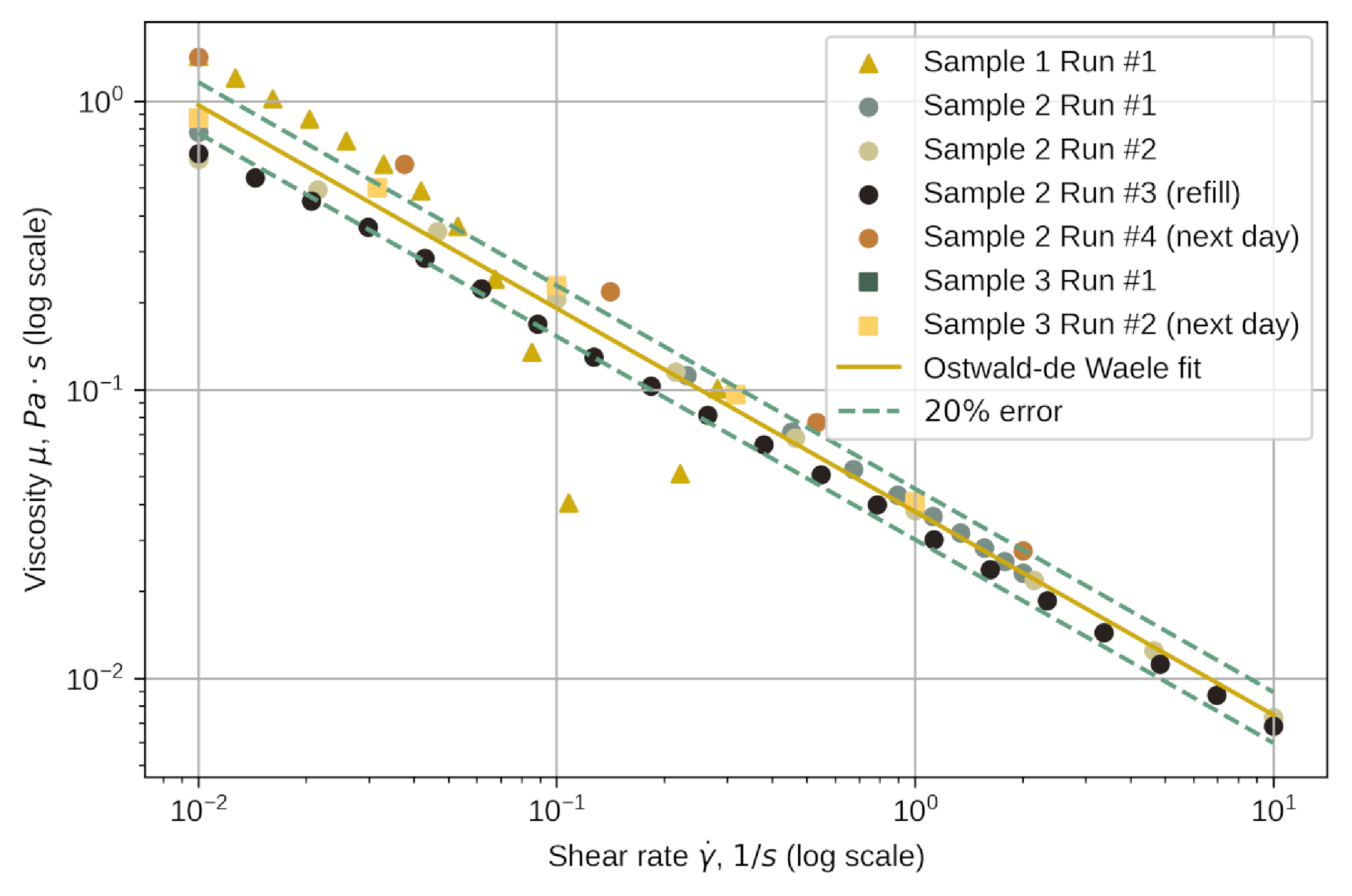

Supplement: S2 Fig — Viscosity measurements data for DMEM + 10 % FBS. Here we have reviewed three samples, performing measurements multiple times (denoted "runs"), including a refill of the same sample and measurements performed on different days. Ostwald-de Waele equation was fit to the entire ensemble of data points. A deviation 20% from the fit line is included as a measure of the repeatability of the results. (TIF) [file pone.0322069.s002.tif]

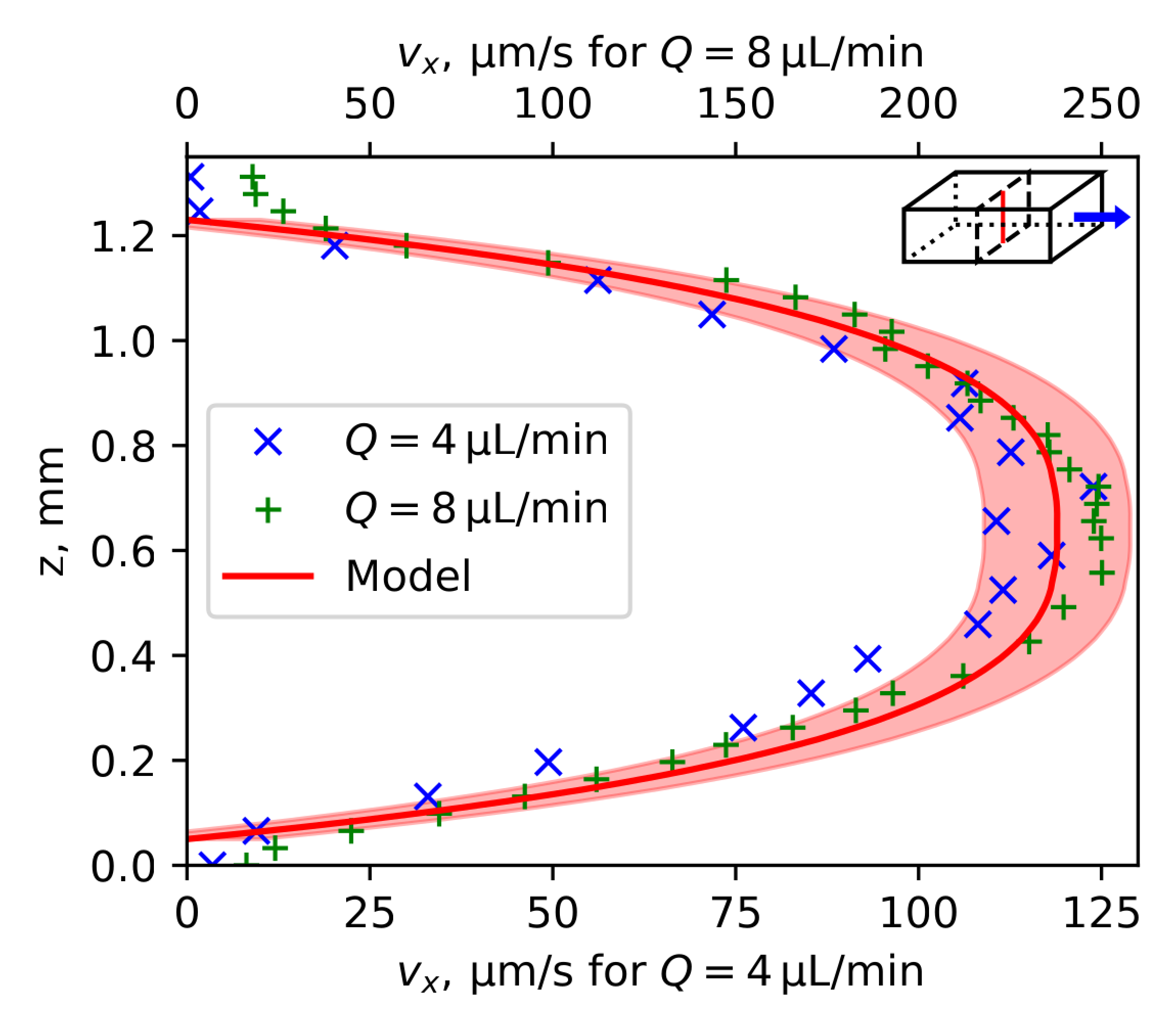

Supplement: S3 Fig — Here we show the experimental velocity values of the DMEM + 1% FBS sample in the middle line of the channel for two different flow rates Q=4 μL/min and Q=8 μL/min. We demonstrate here that increasing the flow rate two times, the fluid velocity increases two times. The model predicts the same shape of the velocity distribution for both flow rates. (TIF) [file pone.0322069.s003.tif]

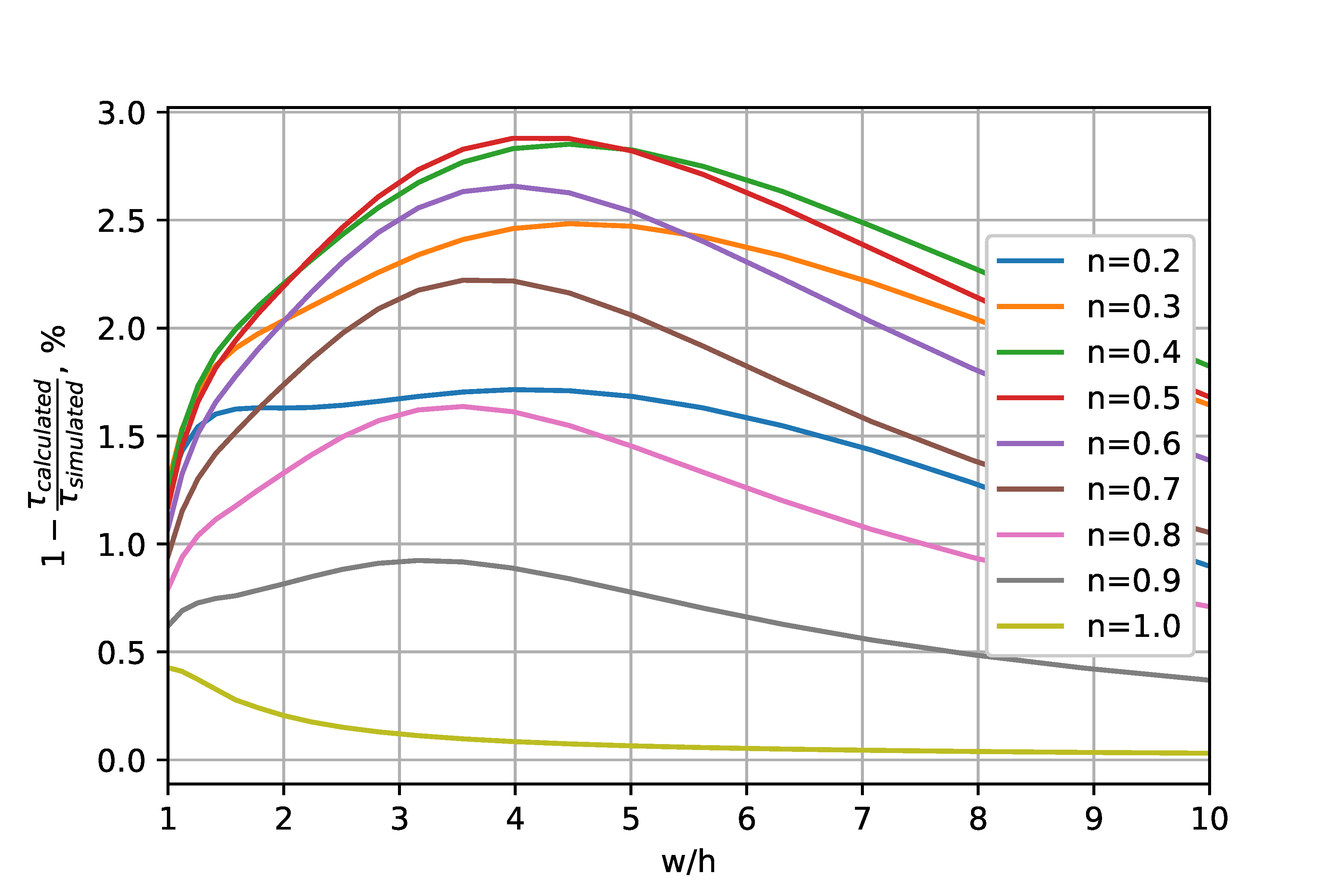

Supplement: S4 Fig — Here we show how large is the error of the proposed formula (1). The graph shows how the shear stress used to calculate flow rate τcalculated using formula (1) differs from shear stress obtained using simulations τsimulated for rectangular channel. The graph shows that shear stress in the channel will be higher than used in the formula by the amount which does not exceed 3%. (TIF) [file pone.0322069.s004.tif]
